# Environmental Programming of Susceptibility and Resilience to Stress in Adulthood in Male Mice

**DOI:** 10.3389/fnbeh.2019.00040

**Published:** 2019-03-01

**Authors:** Catherine Jensen Peña, Eric J. Nestler, Rosemary C. Bagot

**Affiliations:** ^1^Department of Neuroscience and Friedman Brain Institute, Icahn School of Medicine at Mount Sinai, New York, NY, United States; ^2^Department of Psychology, McGill University, Montreal, QC, Canada

**Keywords:** early life stress, depression, development, behavior, stress vulnerability

## Abstract

Epidemiological evidence identifies early life adversity as a significant risk factor for the development of mood disorders. Much evidence points to the role of early life experience in susceptibility and, to a lesser extent, resilience, to stress in adulthood. While many models of these phenomena exist in the literature, results are often conflicting and a systematic comparison of multiple models is lacking. Here, we compare effects of nine manipulations spanning the early postnatal through peri-adolescent periods, both at baseline and following exposure to chronic social defeat stress in adulthood, in male mice. By applying rigorous criteria across three commonly used measures of depression- and anxiety-like behavior, we identify manipulations that increase susceptibility to subsequent stress in adulthood and other pro-resilient manipulations that mitigate the deleterious consequences of adult stress. Our findings point to the importance of timing of early life stress and provide the foundation for future studies to probe the neurobiological mechanisms of risk and resilience conferred by variation in the early life environment.

## Introduction

In the past decade, the chronic social defeat stress (CSDS) paradigm has emerged as one of the most robust and consistent mouse models for depression-like behavioral abnormalities (Berton et al., 2006; Laman-Maharg and Trainor, 2017; Slattery and Cryan, 2017). CSDS induces enduring social avoidance, reminiscent of a hallmark feature of human depression (Berton et al., 2006; Kupferberg et al., 2016; Akil et al., 2017). The translational relevance of CSDS is further supported by the time course of antidepressant response: chronic, but not acute, antidepressant treatment reverses defeat-induced social avoidance, similar to the delayed onset of antidepressant efficacy in humans (Berton et al., 2006). In contrast, single doses of ketamine induce antidepressant-like responses as is also seen in humans (Donahue et al., 2014). Socially defeated mice also exhibit reduced sucrose preference, commonly interpreted as indicating anhedonia, reduced time in the center of an open field, interpreted as an increase in anxiety-like behavior, as well as metabolic and circadian alterations (Krishnan et al., 2007; Lutter et al., 2008; Chuang et al., 2010). Importantly, there is considerable individual variation in social interaction behavior in C57BL/6J mice, with roughly one-third of mice—referred to as resilient—avoiding social avoidance along with all of the other deleterious effects of CSDS except for changes in anxiety-like behavior (Krishnan et al., 2007). Over the past decade, we and other groups have shown that this inherent rate of susceptibility vs. resilience can be shifted in both directions by both molecular and behavioral manipulations, enabling researchers to identify environmental, transcriptional, and neurophysiological factors that bias toward susceptibility or resilience.

**FIGURE 1:**
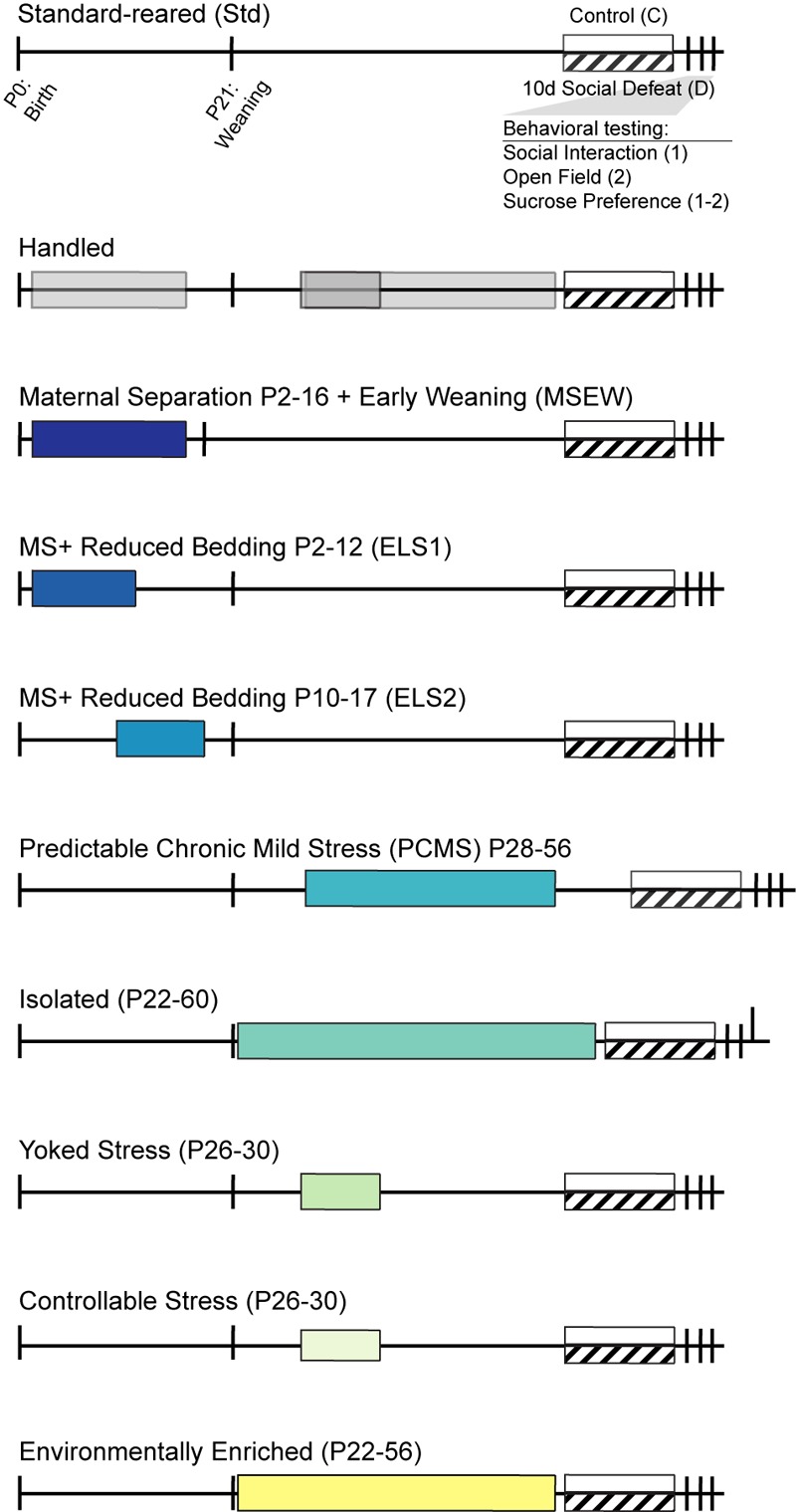
Timeline of early life manipulation models and behavioral testing: Mice were standard-reared (top) or subject to manipulations of the juvenile or peri-adolescent environment at times indicated by shaded bars. Handled mice were included as the sole control for PCMS and Yoked/Controllable manipulations and as an additional control for MSEW mice. In adulthood, mice from each group experienced control conditions (white bar) or 10 days of chronic social defeat stress (hashed bar). Behavioral testing occurred immediately following social defeat.

We sought to determine whether variations in the early life environment from postnatal through peri-adolescent periods could bias behavioral phenotypes toward either susceptibility or resilience to stress in adulthood. Specifically, we set out to establish a mouse “two-hit” model wherein specific early life experience increases susceptibility to depression-like behavior after social defeat in adulthood, and a contrasting model in which different early life experience promotes resilience to the same social defeat in adulthood. Several models of early life adversity, “stress inoculation,” and environmental enrichment have reported baseline changes in anxiety- or depression-like behavior in adulthood, without considering the consequences of exposure to further stress in adulthood. Studies of how early life experience modulates depression- and anxiety-like behavior following a “second hit” of stress in adulthood are lacking. A growing body of epidemiological data in humans suggests that early life stress increases risk for depression and other mood disorders by increasing sensitivity to subsequent stress experienced later in life (Kendler et al., 2004; McGuigan and Middlemiss, 2005; McLaughlin et al., 2017). We have recently reported that one specific early life stress procedure during a defined postnatal period increased susceptibility to adult social defeat stress (Peña et al., 2017). Here, we extend our investigation to four additional early life manipulations conducted at varying ages to establish the specificity of developmental timing and manipulation. We hypothesized that maternal separation with early weaning (MSEW), combined maternal separation and reduced nesting in either the early-to-mid or mid-to-late postnatal periods (ELS1 and ELS2), peri-adolescent isolation, and uncontrollable (yoked) juvenile stress might increase susceptibility to CSDS in adulthood (Figure 1). In addition, we tested four early life manipulations that we hypothesized would promote resilience to adult CSDS: brief early handling, predictable chronic mild stress (PCMS), controllable juvenile stress, and environmental enrichment (Figure 1).

We systematically tested the effects of each of these early life manipulations on a battery of three of the most widely used behavioral tests of depression- and anxiety-like behavior in mice: the social interaction test as a measure of social avoidance, sucrose preference in a two-bottle choice test as a measure of an anhedonia-like behavior, and exploration of an open field as a measure of anxiety-like behavior. We applied three key criteria to evaluate the impact of the manipulations in each behavioral test: (1) Post-defeat behavior is altered relative to a model-specific non-defeated control group; (2) Post-defeat behavior is altered relative to standard-reared defeated mice; or (3) Baseline (pre-defeat) behavior is altered relative to standard-reared control mice. We considered a model to exhibit evidence of increasing susceptibility to a second stress if criteria 1 (significantly altered behavior after social defeat relative to the within-model control) and 2 (significantly altered behavior after social defeat relative to standard-reared defeated mice) were both met. A model that met criterion 3 but not 1 or 2 demonstrated baseline effects of early-life manipulations, but did not model susceptibility to further stress in adulthood. A model that failed to meet all three of these three criteria was considered to promote resilience (i.e., behavior was not modified at baseline nor by defeat), as were manipulations that met criterion 3 by significantly changing behavior in a direction opposing defeat-induced change in standard-reared mice.

## Materials and Methods

### Mice

C57BL/6J mice were maintained on a 12 h light/dark cycle (lights on at 7 am) with *ad libitum* access to food and water. All experiments were conducted in accordance with the guidelines of the Institutional Animal Care and Use Committee at Mount Sinai and the Society for Neuroscience. The protocol was approved by the Institutional Animal Care and Use Committee at Mount Sinai. Home cage bedding, consisting of corn cob material with EnviroDri nesting material, was changed weekly. Adult social defeat and control manipulations occurred during the light cycle in cages with small woodchip bedding to distinguish environment. All behavioral testing occurred in the first half of the light cycle on the days immediately following the end of social defeat stress.

For pre-weaning manipulations, timed pregnant females (Jackson) were ordered to arrive on E14 (MSEW experiment including standard reared and handled controls), or mice were bred in-house (standard-reared, ELS1, ELS2). For in-house breeding, two primiparas females (Jackson) were mated with one male in our animal facility. The male was removed after 1 week and females rehoused in individual cages 1–3 days prior to giving birth (P0). Offspring were weaned at postnatal day P21 into same-sex cages of 3–5 mice, keeping littermates together or combining pups from different litters of the same age and experimental condition to maintain minimum 3 mice/cage.

For manipulations after P21 (PCMS, isolation, controllable or yoked stress, enrichment, cohort standard, and handled controls), C57BL/6J males (Jackson) arrived on P21 and were habituated for 1–5 days prior to manipulation.

### Early Life Paradigms (Figure 1)

#### Standard Facility-Reared (Std)

On the day of birth (P0), litters were weighed and counted and cages cleaned but otherwise undisturbed. Thereafter, cages were cleaned weekly with minimal disruption to the litter. Standard facility-reared mice were generated in each cohort as controls for MSEW (*n* = 8–11), ELS1/ELS2 (*n* = 7 for SI replication; *n* = 12–17 for OFT, sucrose), isolation (*n* = 9–12), and enrichment (*n* = 9–10) experiments.

#### Handling

Handling of mice was performed as an additional control for MSEW mice and as the sole control for PCMS and controllable/yoked mice. For the MSEW model, the dam was removed from the home cage daily from P2-P16, and placed in a separate clean cage with *ad libitum* food and water for 15 min and pups were briefly handled in the dam’s absence (*n* = 11–14). Summary statistics for handled mice are derived from handled vs standard-reared mice within the MSEW model. For the controllable and yoked manipulations, a separate group of mice were handled for 5 days starting at P26 (*n* = 5–10). For the PCMS model, a separate group of mice were handled for 28 days starting at P28 (*n* = 7–10). Peri-adolescent handling consisted of briefly removing mice to a clean cage and returning them to their home cage within 5 min.

#### Maternal Separation With Early Weaning (MSEW)

In a protocol modified from George et al. (2010), the dam was removed from the home cage daily from P2-P16 and placed in a separate clean cage with *ad libitum* food and water. Separations were for 4 h from P2-5 and for 8 h from P6-16. Pups remained together in the home-cage on a heating pad to maintain constant temperature (32–34°C). Pups were weaned on P17 with moistened food pellets placed on the cage floor to ensure adequate nutrition and were checked for dehydration daily for 1 week (*n* = 7–9).

#### Combination Early Life Stress (ELS1 and ELS2)

This paradigm was conducted as described previously (Peña et al., 2017). Briefly, a combination of maternal separation and limited nesting (Gilles et al., 1996; Rice et al., 2008; Molet et al., 2014) was implemented from either P2-12 (ELS1) or from P10-20 (ELS2). Pups were separated together as a litter to clean cages with distinct bedding for 3–4 h/day during the light cycle. ELS1 separation cages were on a heating pad to maintain constant low temperature (32–34°C); heating pads were not used for ELS2 separations as by that age pups are able to thermoregulate on their own. Separations were conducted at random times each day to minimize predictability and habituation. EnviroDri nesting material was depleted to 1/3 of standard-reared cages during the days of separations and then restored. Social interaction behavioral data for these groups are from an independent replication cohort in which only social interaction was assessed (*n* = 10–11). Open field and sucrose preference behavior were from previously reported datasets (*n* = 7–20; Peña et al., 2017), using a restricted open field center consistent with data collected from other models, and statistical analysis after collapsing within litters (see below).

#### Predictable Chronic Mild Stress (PCMS)

In a protocol modified from Suo et al. (2013), mice were subjected to daily, 5 min restraint stress in a 50 mL conical tube between 2 and 3 pm for 28 days, starting at P28. Throughout this period mice were group housed 5/cage. CSDS began 1 week after the last stress (*n* = 9–10).

#### Peri-Adolescent Isolation

Mice were individually housed from P22-60 when social defeat began (*n* = 9–12).

#### Juvenile Controllable and Yoked Stress

To test the hypothesis that controllable stress would promote resilience to later stress, while uncontrollable stress would promote susceptibility (Prince and Anisman, 1990; Drugan et al., 1997; Kavushansky et al., 2006), mice were subject to daily swim stress starting at P26 for 5 days. Mice assigned to the controllable stress group were placed at one end of a large container (30 × 60 cm) filled with 15 cm of warm water (25°C) and with a platform just below the surface at the other end, and upon reaching the platform were removed and gently dried. Five trials at 15-min intervals were conducted each day. As an uncontrollable stress, mice were placed in an identical swim container without a platform, and removed when the yoked partner reached its platform. Mice in the controllable condition learned rapidly within and across days (range of swim duration 1–15 s). Social defeat began approximately 4 weeks after the last swim stress (*n* = 5 controllable and yoked control, 8–10 controllable and yoked defeat).

#### Peri-Adolescent Environmental Enrichment

From P22-56, mice were housed 5/cage in large hamster cages with toys changed weekly, including tubes, a hut, a fast track running wheel, and swing (Lepack et al., 2016). Mice were moved directly from enriched housing into social defeat (*n* = 9–10).

### Adult Chronic Social Defeat Stress

Experiments utilized an established CSDS protocol to induce depressive-like behaviors in male mice (Berton et al., 2006; Krishnan et al., 2007; Golden et al., 2011). Retired breeder CD1 male mice (Charles River) were screened for aggressiveness. Adult (8–10 weeks) mice were subjected to 10 daily, 5-min defeats by a novel CD1 aggressor mouse and were then housed across a perforated plexiglass divider to allow continued sensory contact without further physical aggression for the remainder of each day. Mice were separated sooner than 5 min only if wounding occurred, at which point mice were immediately separated across the barrier. C57BL/6J experimental mice were counterbalanced by early life condition to control for aggressor exposure such that each group was rotated through overlapping sets of aggressor mice. Control mice were housed in cages separated from other control mice by a perforated plexiglass divider and were rotated to a different cage daily. Mice were individually housed in clean cages following the final bout of defeat.

### Social Interaction Test

**FIGURE 2:**
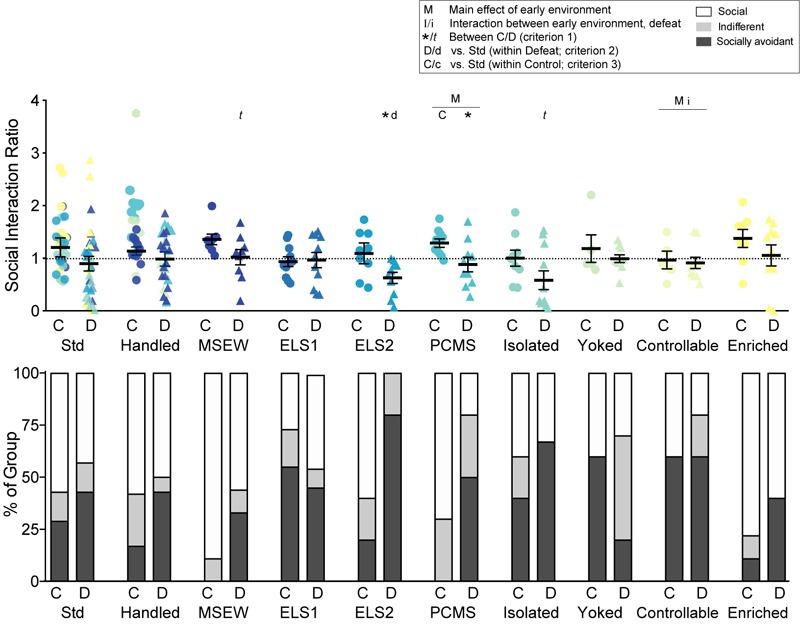
Social interaction: A social interaction ratio was calculated (time spent exploring a novel CD1 mouse / time spent exploring an empty enclosure) for all mice. Dotted line indicates ratio = 1. Individual data points and SEM are shown for each group. Within-model standard-reared or handled groups are overlaid and indicated by model-matched colors; mean and SEM are shown for standard mice from the ELS1/2 cohort, while mean and SEM for handled mice are shown from the MSEW cohort. Proportions of each group classified as social (ratio > 1.1), indifferent (ratio > 0.9 and <1.1), or socially avoidant (ratio < 0.9) are indicated (bottom). C, control conditions; D, social defeat stress. Main effect of early experience manipulation (M *p* < 0.05 and m *p* < 0.1) and interaction between early experience and adult stress (I *p* < 0.05 and i *p* < 0.1) were determined by one-way ANOVA. Significance of criterion 1, within model between C and D conditions, is indicated by: ^∗∗^*p* < 0.01, ^∗^*p* < 0.05 and *t p* < 0.1. Significance of criterion 2, within defeat vs. Std/Handled, is indicated by D *p* < 0.05 and d *p* < 0.1. Significance of criterion 3, within control vs. Std/Handled, is indicated by C *p* < 0.05 and c *p* < 0.1.

Social avoidance behavior, a robust and reproducible measure to distinguish susceptible vs resilient male mice after CSDS (Krishnan et al., 2007), was assessed with a novel CD1 mouse in a two-stage social-interaction test under red lighting as previously described (Berton et al., 2006). In the first 2.5-min test (no target), the experimental mouse was allowed to freely explore an arena (44 cm × 44 cm) containing a plexiglass and wire mesh enclosure (10 cm × 6 cm) centered against one wall of the arena. In the second 2.5 min test (target), the experimental mouse was immediately returned to the arena with a novel CD1 mouse enclosed in the plexiglass wire mesh cage. Time spent in the “interaction zone” (14 cm × 26 cm) surrounding the plexiglass wire mesh cage, “corner zones” (10 cm × 10 cm), and “distance traveled” within the arena was measured by video tracking software (Ethovision, Noldus). A social interaction ratio (SI Ratio, Figure 2, top panel) was calculated as time spent in the interaction zone with the target present vs. target absent. “Socially avoidant” and “social” mice were defined as having an SI Ratio of <0.9 or >1.1, respectively, with remaining mice characterized as “indifferent.”

### Sucrose Preference Test

Sucrose preference, commonly interpreted as a measure of anhedonia-like behavior in mice, was assessed in a home cage two-bottle choice test (Krishnan et al., 2007). Mice were acclimated overnight with two bottles of drinking water (50-mL conical tubes fitted with spouted rubber tops). After social interaction testing, water in one bottle was replaced with a 1% sucrose solution and both bottles weighed. Bottles were weighed again daily at the beginning of the light cycle for 2 days. Bottle locations were switched at each measurement to prevent side bias. Percent sucrose preference was calculated as amount (g) sucrose solution consumed over total amount (g) of water and sucrose consumed.

### Open Field Test

Exploration of an open field arena (44 cm × 44 cm) was assessed during a 10 min test under red lighting. A video-tracking system (Ethovision, Noldus) measured locomotor activity, as well as the time spent in the center (24 cm × 24 cm) of the test arena as an index of anxiety-like behavior.

### Statistical Analysis

All animals from a litter experienced the same early life conditions. Siblings were randomly assigned to different adult conditions. Subject number occasionally varied within a group between outcome measures due to improper video recording or leaked sucrose preference bottles. When multiple offspring from the same litter were included in one group, a single litter-mean was calculated for each outcome measure and used for statistical analysis (MSEW and ELS1/2), although all individual data points are graphed. Outliers, defined by values deviating from the group mean by more than two standard deviations, were excluded.

Prism (version 8, GraphPad) and SPSS (IBM, v25) were used for all graphing and statistical analysis. Significance thresholds were set at *p <* 0.05. Comparisons were calculated based on the within-experiment standard-reared group, except for PCMS and controlled/yoked mice which were compared to their handled counterparts. Figures are presented together by overlaying standard-reared and handled mice across models in order to facilitate comparisons. There were no significant main effects of cohort among standard-control groups for SI ratio or sucrose preference, but there were main effects of cohort on open field measures. Main effects and interactions were analyzed by two-way ANOVA. Two-tailed Student’s *t-*tests were used for explicit comparisons between two groups at a time to evaluate each of the three pre-established criteria. Cohen’s D was calculated in Excel from SPSS output as a measure of effect size for these comparisons. Differences between proportions of social and socially avoidant mice were assessed by Chi-square test; for ease of interpretation, statistics reported restrict consideration to social and socially avoidant animals (i.e., excluding “indifferent”), however, similar values were obtained when indifferent animals were included. Effect size for proportional comparisons were taken from attributable risk calculations (P1-P2; Prism 8).

## Results

### Social Avoidance

We calculated a social interaction ratio (Figure 2, top panel) as a within-animal measure of social avoidance and widely used assessment of depression-like behavior and of susceptible vs resilience after CSDS. There was a trend for an interaction between early life experience and adult stress for controllable stress compared to handling (*F*_1,26_ = 4.086, *p* = 0.054). There was also a main effect of PCMS (*F*_1,35_ = 4.315, *p* = 0.045) and of controllable stress (*F*_1,26_ = 4.628, *p* = 0.041). There was a main effect (*p* < 0.05) of adult defeat on SI ratio within seven models including handled, MSEW, ELS2, PCMS, isolation, yoked, and controllable models, and a trend for a main effect of defeat among enriched mice (*p* = 0.093). Defeat decreased SI ratio relative to non-defeated controls (criterion 1) in two early experience models: ELS2 (*t*_1,12_ = 2.436, *p* = 0.031) and PCMS (*t*_1,18_ = 2.566, *p* = 0.019), with trends observed in MSEW (*t*_1,11_ = 1.828, *p* = 0.095) and isolated (*t*_1,18_ = 1.742, *p* = 0.099) groups. Although no defeated group showed a significant reduction in SI ratio compared to standard-reared defeated mice (criterion 2), this effect was trending in ELS2 defeated mice (*t*_1,9_ = 2.118, *p* = 0.063). Among control, non-defeated mice, only PCMS significantly decreased SI ratio relative to handled mice (criterion 3; *t*_1,18_ = 2.661, *p* = 0.016).

We next analyzed the proportions of mice exhibiting social, indifferent, or socially avoidant behavior after CSDS based on SI ratio (Figure 2, lower panel). Defeat significantly changed the proportion of social vs socially avoidant mice among all models (*p* < 0.05 for Std, ELS1, isolated, yoked, and controllable models; *p* < 0.01 for handled, MSEW, ELS2, PCMS, and enriched models; criterion 1). Among defeated mice, the proportion of socially avoidant mice was reduced among yoked mice (*X*^2^ = 6.268, *p* = 0.012) and increased among ELS2 (*X*^2^ = 53.98, *p* < 0.001), PCMS (*X*^2^ = 18.53, *p* < 0.001), isolated (*X*^2^ = 15.11, *p* < 0.001), yoked (*X*^2^ = 6.27, *p* = 0.012), and enriched (*X*^2^ = 8.00, *p* = 0.005) relative to standard-reared mice (criterion 2). Among non-defeated control mice, the proportion of socially avoidant mice was significantly reduced among handled and MSEW mice (*X*^2^ > 16.53, *p* < 0.001; criterion 3), whereas ELS1, yoked, and controllable models proportionally increased social avoidance (*X*^2^ > 18.68, *p* < 0.001).

### Sucrose Preference

**FIGURE 3:**
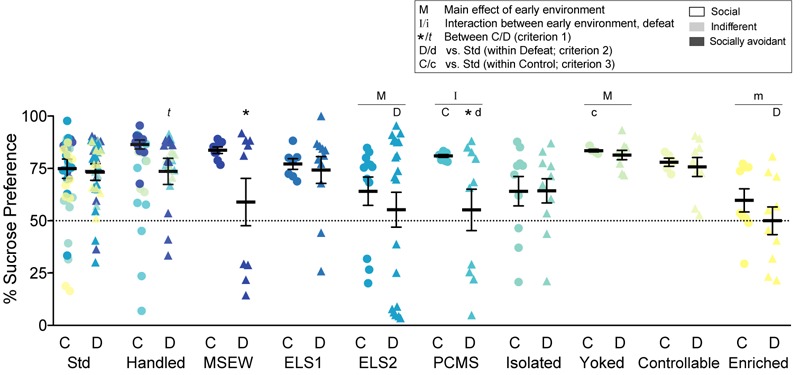
Sucrose preference: Percent preference for a 1% sucrose solution vs. water. Individual data points and SEM are shown for each group. Within-model standard-reared or handled groups are overlaid and indicated by model-matched colors; mean and SEM are shown for standard mice from the ELS1/2 cohort, while mean and SEM for handled mice are shown from the MSEW cohort. Dotted line indicates chance choice levels at 50%. C, control conditions; D, social defeat stress. Within model between C and D conditions: *t* indicates *p* < 0.1. Main effect of early experience manipulation (M *p* < 0.05 and m *p* < 0.1) and interaction between early experience and adult stress (I *p* < 0.05 and i *p* < 0.1) was determined by one-way ANOVA. Significance of criterion 1, within model between C and D conditions, is indicated by: ^∗^*p* < 0.05 and *t p* < 0.1. Significance of criterion 2, within defeat vs. Std/Handled, is indicated by D *p* < 0.05 and d *p* < 0.1. Significance of criterion 3, within control vs. Std/Handled, is indicated by C *p* < 0.05 and c *p* < 0.1.

We calculated sucrose preference (Figure 3), a widely used assay of depression-like behavior commonly interpreted as a measure of anhedonia. There was a significant interaction between early life experience and chronic adult social defeat stress for PCMS (*F*_1,32_ = 9.5663, *p* = 0.004). There was a main effect of early life experience among ELS2 (*F*_1,51_ = 5.595, *p* = 0.021) and yoked (*F*_1,25_ = 5.654, *p* = 0.0*25*) models as well as a trend among enriched (*F*_1,34_ = 3.407, *p* = 0.074) mice. There was also a main effect of defeat among MSEW mice (*F*_1,24_ = 5.113, *p* = 0.033) with a trend among handled mice (*F*_1,27_ = 3.924, *p* = 0.058). Mean sucrose preference was significantly lowered among defeated MSEW (*t*_1,12_ = 2.219, *p* = 0.047) and PCMS (*t*_1,17_ = 2.464, *p* = 0.025) mice, with a trend among handled mice (*t*_1,15_ = 2.013, *p* = 0.077), compared to their control counterparts (criterion 1). Among defeated mice (criterion 2), sucrose preference was decreased by ELS2 (*t*_1,29_ = 2.155, *p* = 0.040) and enrichment (t_1,17_ = 2.724, *p* = 0.014) relative to their standard-reared counterparts, and moderately decreased by PCMS (*t*_1,16_ = 1.958, *p* = 0.068) relative to their handled counterparts. Among control mice (criterion 3), sucrose preference was elevated by PCMS (*t*_1,16_ = 2.457, *p* = 0.038) and, to a lesser extent, among yoked mice (*t*_1,8_ = 2.121, *p* = 0.098). Individual differences in sucrose preference did not correlate with social interaction suggesting that these behavioral phenotypes assess dissociable aspects of depression-like behavior that are differentially regulated by stress across the lifespan (not shown).

### Anxiety-Like Behavior in an Open Field

**FIGURE 4:**
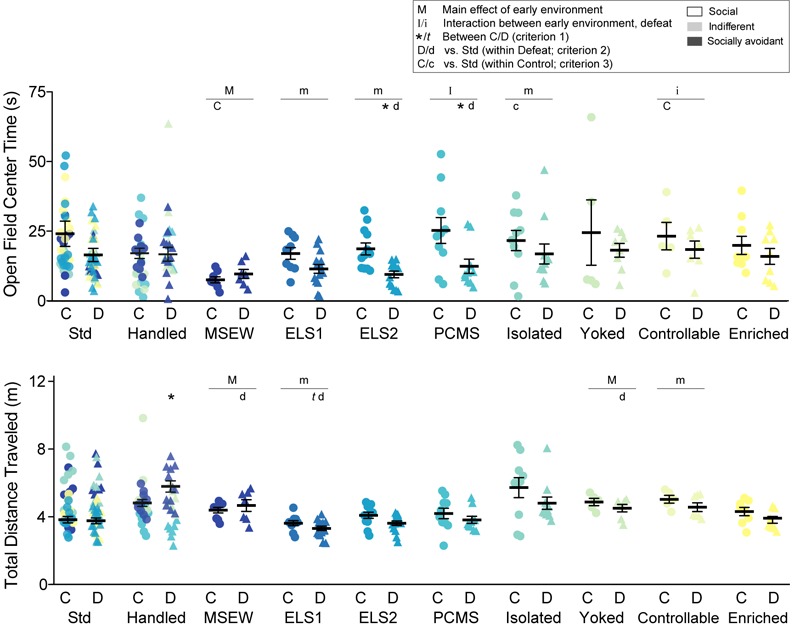
Open field: Time spent exploring the center of a novel open field (top panel) and total distance traveled over 10 min (bottom panel). Individual data points and SEM are shown for each group. Within-model standard-reared or handled groups are overlaid and indicated by model-matched colors; mean and SEM are shown for standard mice from the ELS1/2 cohort, while mean and SEM for handled mice are shown from the MSEW cohort. C, control adult conditions; D, social defeat stress. Main effect of early experience manipulation (M *p* < 0.05 and m *p* < 0.1) and interaction between early experience and adult stress (I *p* < 0.05 and i *p* < 0.1) was determined by one-way ANOVA. Significance of criterion 1, within model between C and D conditions, is indicated by: ^∗^*p* < 0.05 and *t p* < 0.1. Significance of criterion 2, within defeat vs. Std/Handled, is indicated by D *p* < 0.05 and d *p* < 0.1. Significance of criterion 3, within control vs. Std/Handled, is indicated by C *p* < 0.05 and c *p* < 0.1.

We measured time in the center of an open field (Figure 4, top panel), a widely used index of anxiety-like behavior. There was an interaction between PCMS and adult stress (*F*_1,34_ = 4.678, *p* = 0.038), and a trend for an interaction with controllable peri-adolescent stress (*F*_1,22_ = 3.027, *p* = 0.096), on open field center time. There was a main effect of MSEW (*F*_1,21_ = 8.053, *p* = 0.010) and trends for main effects of ELS1, ELS2, and isolated models (*p* < 0.1). There was also a main effect (*p* < 0.05) of adult chronic social defeat on center time among ELS1, ELS2, and enriched models. Adult CSDS significantly decreased center time relative to within-model controls (criterion 1) ELS2 (*t*_1,18_ = 3.106, *p* = 0.006) and PCMS (*t*_1,18_ = 2.243, *p* = 0.026). Among defeated mice, open field center time was not significantly reduced by any early manipulation relative to their standard or handled within-model counterparts (criterion 2), although there were trends with ELS2 (*t*_1,16_ = 1.992, *p* = 0.064) and PCMS (*t*_1,16_ = 1.866, *p* = 0.080). Among control, non-defeated mice (criterion 3), open field center time was increased by controllable peri-adolescent stress (*t*_1,8_ = 2.619, *p* = 0.031) and to a lesser extent by isolation (*t*_1,15_ = 1.889, *p* = 0.078) and decreased by MSEW (*t*_1,11_ = 2.582, *p* = 0.026).

We also measured total distance traveled in the arena during the 10-min test which may reflect differences in habituation to a novel environment (Figure 4, lower panel). While no animals exhibited outwardly observable locomotor impairments, there was a main effect of early life manipulation on distance traveled among MSEW (*F*_1,22_ = 5.636, *p* = 0.027) and yoked models (*F*_1,23_ = 4.59, *p* = 0.043), and trending main effects of ELS1 and controllable peri-adolescent stress. There were also trending main effects (*p* < 0.1) of defeat among handled, PCMS, isolated, yoked, controllable, and enriched models. CSDS increased total distance traveled among handled mice (*t*_1,15_ = 2.609, *p* = 0.020) and trended to decrease distance traveled among ELS1 (*t*_1,17_ = 1.775, *p* = 0.094) mice relative to their within-model controls. Among defeated mice (criterion 2), no model significantly altered total distance traveled relative to their standard or handled within-model counterparts, although there were trends for decreased distance traveled among MSEW (*t*_1,10_ = 1.982, *p* = 0.076), ELS1 (*t*_1,18_ = 1.937, *p* = 0.069), and yoked (*t*_1,15_ = 1.832, *p* = 0.086) models. No models significantly altered distance traveled among control mice relative to within-model standard or handled counterparts.

Given alterations in distance traveled, we calculated a ratio of center time/total distance traveled for all models. There was an interaction among PCMS mice, a main effect of ELS2, and main effects of defeat among ELS1 and ELS2 models (*p* < 0.5, not shown), similar to analysis of open field center time.

## Discussion

We sought to establish the specificity of a mouse “two hit” stress model that enhances stress susceptibility in adulthood, and to develop a model whereby early experience promotes resilience in the face of chronic stress in adulthood. To this end, we systematically examined the delayed effect of nine environmental manipulations, spanning the early postnatal, late postnatal, and peri-adolescent periods, in altering responses to CSDS in adulthood. Susceptibility and resilience were assessed by a battery of three widely used tests of depression- and anxiety-like behavior with all experiments conducted under comparable conditions in the same animal facility to facilitate direct comparison across manipulations, eliminating a source of variation that has confounded efforts to synthesize effects of early life manipulations among published studies. We assessed behavior within each model in a standardized test battery applied against three independent criteria to systematically evaluate the phenotype induced by each manipulation: *criterion 1*: model control and model defeated mice are significantly different from each other; *criterion 2*: model defeated mice are significantly different from standard-reared defeated mice; *criterion 3*: model mice not subjected to defeat are significantly different from standard-reared control mice. A model was considered to increase susceptibility if it met criteria 1 and 2 and was considered to promote resilience if it failed to meet criteria 1–3 or induced significant differences in the opposite direction from defeat in standard-reared mice.

**FIGURE 5:**
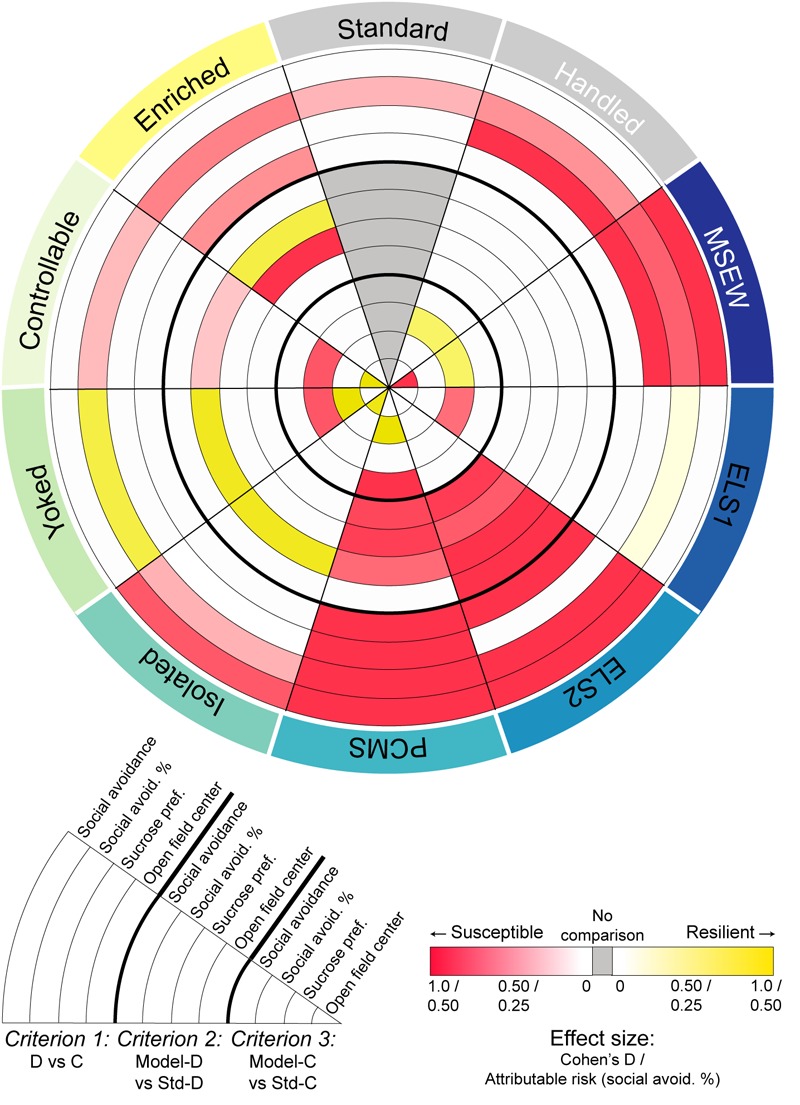
Effect size across statistical analyses: Integrated comparison of behavioral findings across models for all comparisons reaching at least trending (*p* < 0.1) significance. Red indicates effect size when group mean shifted in a pro-susceptible direction; yellow indicates effect size when group mean shifted in a pro-resilient direction; gray indicates no comparison possible. Effect size is represented by Cohen’s D for all comparisons except proportional analyses which are represented by attributable risk values. Pie slices are nested by behavioral test and clustered by the three criteria employed for determining susceptibility or resilience.

The effects of most early life manipulations on depression- and anxiety-like behavioral tests after CSDS in adulthood were more modest than anticipated. Figure 5 integrates the statistical assessment of the three criteria for increasing susceptibility or promoting resilience across behavioral tests. We originally hypothesized that MSEW, ELS2, and uncontrollable yoked stress would all increase susceptibility to depression-like behavior after chronic stress in adulthood, and that handling, PCMS, controllable stress, and enrichment would increase resilience. Consistent with our hypothesis, we show that ELS2 increases susceptibility to a second hit of stress by the current criteria. Somewhat unexpectedly, PCMS, a manipulation previously reported to promote resilience (Suo et al., 2013), also increased susceptibility, and isolation, yoked stress, and enrichment induced a partial pro-resilience phenotype. These findings are discussed in more detail below.

We validated the social interaction results of our previous findings that ELS2 increases susceptibility to a second hit of stress (Peña et al., 2017). ELS2 met both criteria 1 and 2 for susceptibility, inducing lower SI ratio and open field center time in mice exposed to both stressors than mice exposed to either stress alone. ELS2 also met criterion 2 for sucrose preference. The specificity of this finding is established by the comparison to other models. For example, ELS1, a manipulation identical to ELS2 but conducted in the early postnatal period—a time when a majority of rodent early life stressors have been imposed—failed to meet criterion 1 or 2 for any test, although there was a main effect of ELS1 in the open field test. These results suggest a late postnatal sensitive period during which stress can increase sensitivity to later stress in adulthood. This is consistent with findings that the first 10 days of rodent life are a relatively stress-insensitive period that favors caregiver attachment irrespective of prevailing conditions (Rincón-Cortés and Sullivan, 2014). Between P10-16, pups transition out of this stress hyporesponsive period, endogenous corticosterone levels increase, and pups acquire the ability to form amygdala-dependent fear associations that can be substantially buffered by the mother’s presence (Moriceau and Sullivan, 2006).

MSEW was developed as a mouse model of early life neglect (George et al., 2010; Carlyle et al., 2012). Previous maternal separation studies with or without early weaning found mixed effects of MSEW on open field center time in adulthood, increased immobility in a forced swim test, decreased sucrose preference, and some changes in brain connectivity (George et al., 2010; Carlyle et al., 2012; Amiri et al., 2016). We therefore hypothesized that MSEW would increase depression-like behavior after a second hit of stress in adulthood. While MSEW met criterion 1 in the sucrose preference test, it also met criterion 3 in the open field test indicating baseline effects, and overall it did not robustly increase susceptibility to stress in adulthood. The MSEW paradigm temporally overlapped with the same putative stress-sensitive period as ELS2, indicating that not all stress encountered in this period is equal. One difference between these procedures is that MSEW removed the dam from the home cage (with pups remaining in the home cage), whereas ELS1 and ELS2 removed the pups to clean cages. The maternal and home cage odors may therefore have buffered the stress response through P16 among MSEW pups, limiting the impact of this manipulation (Moriceau and Sullivan, 2006). We hypothesize, although it remains to be tested, that altering the MSEW protocol to remove pups rather than the dam from the home cage would lead to stronger behavioral changes.

Play with peers is an important driver of social and neurobiological development for humans and rodents alike. Social play peaks between P26-40 (Panksepp, 1981). Post-weaning or peri-adolescent social isolation deprives animals of this age-typical experience and the deleterious effects of peri-adolescent isolation cannot be fully rescued by subsequent resocialization, in contrast to the effects of adult isolation (Einon and Morgan, 1977). We found a trend for social avoidance after defeat among isolated mice, supporting criterion 1 and consistent with previous reports (Hermes et al., 2011). However, the trends for effects supporting criterion 3 and for a main effect of isolation to increase open field center time is inconsistent with our hypothesis of increased susceptibility (Arakawa, 2003). Thus, across behavioral tests, social isolation did not robustly increase susceptibility to stress in adulthood.

Brief handling of pups by an investigator has been generally reported to decrease acutely measured stress responsivity in adulthood relative to standard facility reared rodents for review, see Pryce et al. (2005). We therefore predicted handling would increase resilience to depression-like behavior after CSDS. In support of this hypothesis, handling moderately decreased the proportion of socially avoidant control mice (criterion 3). However, handling did not alter behavior after social defeat on any of the tests relative to standard-reared mice, and did not robustly promote resilience.

Most people who experience early adversity do not develop depression or anxiety and, paradoxically, some studies report that early adversity can even be protective in the face of later adversity, a phenomenon termed “stress inoculation” (Brockhurst et al., 2015; Santarelli et al., 2017). It is theorized that individuals might learn to cope with challenges in a moderately stressful early environment, leaving them better prepared for a later high-threat environment (Chaby et al., 2015). Similarly, the ability to control stress is thought to promote positive stress coping responses, while an inability to control stress leads to learned helplessness and other deleterious consequences (Seligman and Maier, 1967; Drugan et al., 1997). Prior learned ability to control a stress can also attenuate response to a later uncontrollable stress (Maier and Watkins, 2005; Amat et al., 2006; Baratta et al., 2007). We therefore hypothesized that predictable, mild stress or controllable stress in the peri-adolescent period would protect against stress-induced depression-like behavior in adulthood, while yoked uncontrollable stress might instead increase subsequent susceptibility. While sucrose preference under control conditions was indeed increased by PCMS, overall PCMS met criteria 1 and 2 for *increasing* susceptibility to a second hit of stress. It was also surprising that yoked stress promoted resilience in terms of decreasing the proportion of socially avoidant mice, and that controllable stress had little effect on susceptibility. Both controllable and yoked foot-shock stress were previously found to induce similar corticosterone responses (Prince and Anisman, 1990), suggesting that the consequences of peri-adolescent stress may be directed primarily by hormonal mediators rather than perceived stressor controllability. The time course of stress manipulations may also have contributed to the differential effects of controllable and yoked stress vs PCMS. While all stressors started at the same age, controllable and yoked stress concluded after 5 days with 4 weeks recovery prior to defeat stress, whereas PCMS continued for 28 days with only 1 week recovery prior to the second-hit stress. Whether an abridged 5 days of the brief restraint used in PCMS- or a longer recovery period- would also promote resilience is yet to be tested.

Environmental enrichment is a common paradigm for ameliorating depression-like behavior in rodents (Brenes et al., 2008; Cymerblit-Sabba et al., 2013). The protective effects of enrichment are independent of its effects on hippocampal neurogenesis (van Praag et al., 1999; Meshi et al., 2006), and may be mediated by a host of other neurobiological changes including altered levels of the transcription factor cyclic adenosine monophosphate (cAMP) response element binding protein (CREB), and protective epigenetic regulatory mechanisms in the reward circuitry (Green et al., 2010; Lepack et al., 2016). Environmental enrichment promoted resilience by some behavioral measures (proportion of mice exhibiting social behavior relative to standard-reared defeated mice, consistent with Lehmann and Herkenham, 2011; criterion 2), but also, surprisingly, increased susceptibility indicated by other measures in this cohort (sucrose preference, criterion 2). Previous reports on the effects of enrichment on sucrose preference are in fact mixed (Brenes and Fornaguera, 2008; Mileva and Bielajew, 2015). Consistent with our finding of decreased sucrose preference, decreased sucrose-reinforced operant responding and decreased self-administration of amphetamine and cocaine were previously reported for male and female rats exposed to post-weaning environmental enrichment (Green et al., 2010), although enriched rats displayed increased place preference for cocaine. It is possible that decreased sucrose consumption is due to increased sensitivity to rewards, and thus for enriched rodents is not reflective of increased anhedonia or depression-like behavior.

Of note, early environment and CSDS rarely shifted sucrose preference toward chance levels (50%) but instead created an apparent preference for water or possible sucrose aversion in a subpopulation of mice (Figure 3). It is difficult to know if this is typical since many studies report group means rather than individual data points. Individual differences in sucrose preference did not correlate with social interaction (not shown). While sucrose preference is a widely used test of “depression-like” behavior, enthusiasm for this test is perhaps driven more by its simplicity of execution than any inherent validity. A difference in preference is commonly interpreted as indicating anhedonia and disruption of reward systems, however, other interpretations are possible. For example, a non-specific shift in perceptual thresholds for flavor detection is equally plausible and has been reported in human depression (reviewed by Heath et al., 2006, but see Potts et al., 1997 for another interpretation). Interestingly, individuals diagnosed with major depression fail to show expected deficits in a sweet taste test, a human analog of rodent sucrose preference tests (Dichter et al., 2010). It is therefore important for the field to validate other measures of anhedonia-like behavior, perhaps through the use of operant-based tests that assess reward-related biases in decision-making.

The present study has several limitations, the most important of which is that this work was limited to male C57BL/6J mice. We initially set out to identify environmental manipulations to shift susceptibility or resilience to adult stress, choosing the CSDS model based on robust evidence that this model is sensitive to environmental, pharmacological, and neurobiological manipulations to increase susceptibility or resilience (Krishnan et al., 2007). Since these experiments were conducted there have been several reports of implementation of social defeat in female C57BL/6J using experimental manipulations to force female-directed aggression that is not observed in naturalistic interactions (Harris et al., 2017; Takahashi et al., 2017). However, it remains an open question as to whether resulting behavioral phenotypes can be bidirectionally modulated. Additional studies are needed to extend the present findings to determine their relevance to the effects of early life stress and adult stress in female mice, especially given the profound sex differences seen in stress responses in animals and depression in humans (Hodes et al., 2016; Jašarević et al., 2016; Labonté et al., 2017; Seney et al., 2018).

This study did not directly test whether limited bedding/nesting from P2-9 (Gilles et al., 1996; Walker et al., 2017) altered depression-like behavior after a second hit of stress in adulthood. Reduced nesting was incorporated into the ELS1 and ELS2 paradigms, but all cages had standard amounts of corncob bedding in the home cage. Previous studies examined whether limited bedding affected behavior after social defeat in adolescence (Hsiao et al., 2016) or adulthood (Santarelli et al., 2017). Adolescent social defeat after limited bedding increased social interaction and resilience after adolescent social defeat, which the authors attributed to increased stress coping (Hsiao et al., 2016). In contrast, adult social defeat after limited bedding increased measures of depression-like behavior (Santarelli et al., 2017), indicating that the timing of the second stress is important.

Early life stress, such as child abuse, neglect, or death or incarceration of a parent, increases risk of a later psychiatric diagnosis (Heim and Nemeroff, 2001; Kessler et al., 2010; Andersen, 2015). However, only a minority of people who experience early life adversity will experience a mood disorder at some point in their lifetime. Human and animal research suggests that early life adversity elevates risk by amplifying sensitivity to stress experienced later in life (Kendler et al., 2004; McGuigan and Middlemiss, 2005; Zhang et al., 2016; Asselmann et al., 2018). Establishing a robust mouse model of susceptibility to stress in adulthood is an essential foundation upon which to probe the causal neurobiological mechanisms by which early experience sensitizes individuals to subsequent stress and depression-like behavior (Peña et al., 2017). Our systematic behavioral methodology revealed that ELS2 and PCMS meet criteria for increasing susceptibility to a second hit of stress across multiple behavioral tests. In contrast, yoked stress in the juvenile period somewhat promoted resilience to adult stress by the current criteria.

Our results enable evaluation of several competing hypotheses of how early adversity affects later stress responses. According to the “cumulative stress hypothesis,” stress effects across the lifespan accumulate and mood disorders present upon reaching a critical threshold of combined stress (Vinkers et al., 2014). This is sometimes held in contrast to a “stress sensitive period hypothesis” (Björkenstam et al., 2017). In addition, a “stress mismatch hypothesis” postulates that stress experienced early in life prepares an individual for later stress such that an individual’s fitness is highest if the adult environment matches their early life environment, and lowest if there is a mismatch (low stress early but high stress in adulthood, or vice versa) (Bagot et al., 2009; Daskalakis et al., 2012; Van Camp et al., 2018). Broadly, we find that stress effects were not strictly additive, as not all early life stressors increased depression- and anxiety-like behavior and additional adult stress only exacerbated behaviors in some models. Our current behavioral tests also find limited evidence for a stress-matching hypothesis, as only yoked stress produced maladaptive behaviors alone (proportion of socially avoidant mice; criterion 3) that were ameliorated upon experience of adult social defeat stress. Our finding that ELS2, but not ELS1, mediated susceptibility to stress in adulthood supports a stress sensitive period model (Schalinski et al., 2016), whereby stress in the late postnatal period, but not in the early postnatal period, increased susceptibility to stress in adulthood.

To facilitate comparison of a large number of early life manipulations and benchmark our findings to the existing literature, we employed three widely used behaviors associated with depression- and anxiety-like states. However, caution is required in interpreting the immediate relevance of such findings to human major depression. While much data demonstrate that the measured behaviors involve brain systems implicated in depression (e.g., nucleus accumbens, amygdala, prefrontal cortex, hippocampus), the basis of their wide use is due more to their procedural simplicity than any inherent validity and, alone, such tests cannot fully recapitulate the complexity of the highly heterogeneous syndrome of human depression. For example, depression also impacts learning and memory as well as decision making processes that are beyond the purview of these tests. It will be important for future studies to expand the scope of behavioral assessment to probe more subtle, and potentially translational, metrics of the interplay of early life experience and stress in adulthood.

## Author Contributions

CP, EN, and RB designed the study. CP and RB performed the experiments and wrote the manuscript. CP performed the data analysis. All authors took part in interpretation of the results. EN edited the manuscript.

## Conflict of Interest Statement

The authors declare that the research was conducted in the absence of any commercial or financial relationships that could be construed as a potential conflict of interest.
